# Enhancing Surface Activation in Unidirectional Carbon Fiber/Epoxy Composites through Low-Temperature Plasma

**DOI:** 10.1021/acsomega.5c11555

**Published:** 2026-01-29

**Authors:** Carlos E. Moraes, Fillip C. Alves, Lucas C. da Silva, Luis F. B. Marques, Rogerio P. Mota, Michelle L. Costa, Edson C. Botelho

**Affiliations:** † School of Engineering and Sciences of Guaratinguetá (FEG), São Paulo State University (UNESP), Avenida Doutor Ariberto Pereira da Cunha, 333, Pedregulho, Guaratinguetá, SP 12516-410, Brazil; ‡ Forza Composites, Avenida Shishima Hifumi, 2911, Urbanova, São José dos Campos, SP 12244-000, Brazil

## Abstract

The physicochemical characteristics of carbon fiber reinforced polymer (CFRP) surfaces are critical for structural bonding. Achieving optimal adhesion remains challenging, as conventional treatments often involve aggressive chemicals or toxic residues. Plasma treatments offer an environmentally friendly alternative, inducing surface modifications such as increased roughness, enhanced hydrophilicity, incorporation of high-energy functional groups, and reactive sites formation. This study investigates argon, nitrogen, and oxygen plasma treatments on unidirectional carbon fiber/epoxy composites (CYCOM 5320-1) at varying power (20 and 50 W) and durations (600, 1200, 1800 s) to evaluate effects on surface activation, hydrophobic recovery, temporal stability, and bulk viscoelastic properties. Results show plasma effectively cleans and decontaminates surfaces while improving roughness and wettability. Argon plasma at low power and short duration increased roughness by ∼ 17% and enhanced existing functional groups. Nitrogen plasma at low power and longer durations increased roughness by 140% and introduced nitrogen-rich groups, improving wettability. Oxygen plasma produced the most substantial effects even at low power, with ∼200% roughness increase and incorporation of highly energetic oxygen-containing groups, favorable for structural adhesion. FTIR, XPS, SEM, and DMA analyses supported these findings, providing detailed insights into surface activation mechanisms.

## Graphical abstract

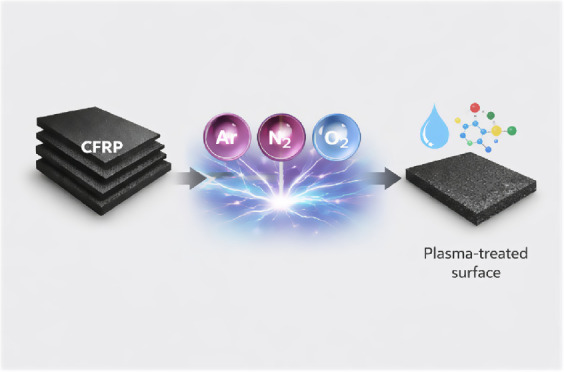

## 1 Introduction

The ongoing pursuit of reducing structural weight while maintaining mechanical performance has made composite materials, especially CFRP, widely utilized in several industrial sectors, including automotive, aerospace, aeronautics, and oil and gas fields.^1–5^ These materials offer several advantages, including easy processing and molding, resistance to impact and fracture, low density, high strength and stiffness, excellent elasticity, long service life, and good corrosion resistance.^6,7^ Despite the benefits, significant challenges persist, particularly in joining methods, highlighting the urgency of advancing reliable connection technologies for composite-to-composite materials due to their increasing use in modern mechanical manufacturing.^8^ Adhesive bonding has become a widely adopted solution mainly due to its ability to join both similar and dissimilar materials without damaging the adherend. This technique offers the advantage of distributing mechanical stresses over a larger area, thereby mitigating the risk of structural deficiencies. Moreover, it eliminates the need for drilling holes, a process that can lead to critical issues in composites, such as fiber breakage, stress concentration, and delamination.^8–10^ Consequently, enhancing adhesion properties has become a prominent research focus, with numerous studies^11–15^ exploring strategies to address this issue. Surface treatments enhance adhesion by creating a contaminant-free surface and increasing its free energy, thereby promoting stronger bonding.^16^ This involves altering the surface topography to improve mechanical interlocking or modifying the chemical composition of the adherend to increase chemical bond affinity at the interface region.^17^ Techniques such as sanding, blasting, surface coating modifications, nanofiller incorporation, and plasma-based surface modification have been extensively employed.^18–21^ Among these, plasma surface modification techniques stand out due to their versatility and efficiency. These methods range from pure plasma ion implantation to hybrid approaches combining ion implantation and deposition, as well as plasma film deposition with substrate biasing.^22^ Such treatments modify material properties without altering their structural characteristics. They can be broadly categorized into: (i) direct chemical surface modifications and (ii) processes inducing both physical and chemical interactions. By ionizing gases such as Nitrogen (N2), Argon (Ar), and Oxygen (O2), high-energy ions and electrons interact with the composite surface, inducing chemical and physical modifications. These changes enhance surface roughness, wettability, mechanical interlocking, and the formation of reactive species that improve chemical adhesion.^23,24^ Low-temperature plasma treatment offers significant advantages as an eco-friendly alternative to conventional surface treatment methods, primarily due to its minimal use of aggressive chemical reagents, low consumption of organic solvents, and elimination of multistep purification processes typical of wet chemical treatments.^25^ This treatment utilizes electron energy and plasma nonequilibrium, enabling the modification of heat-sensitive materials while preserving their structural integrity. Furthermore, this technique ensures controlled and efficient surface modifications in a faster and cleaner manner, with minimal waste generation.^23,26^ Building upon these advantages, low-pressure radio frequency plasmas have been widely explored as an efficient approach for tailoring polymer surface properties, offering precise control over functionalization, wettability, and surface morphology.^27^ Among the available configurations, capacitively coupled RF reactors (RF-CCP, 13.56 MHz) are particularly attractive due to their operational stability at low power and their ability to generate reactive species capable of promoting surface activation. Under typical operating conditions reported in the literature (pressures around 100 mTorr and applied powers in the range of 20–50 W), these discharges exhibit electron temperatures of a few electronvolts and electron densities on the order of 10^9^–10^11^ cm^–3^, which are sufficient to sustain excitation, dissociation, and ionization processes responsible for polymer surface modification.^27,28^ A comprehensive understanding of plasma–surface interaction mechanisms, however, requires detailed characterization of the plasma physicochemical properties. In this regard, diagnostic studies of low-pressure Ar, N_2_, and O_2_ plasmas reported in the literature^29–31^ provide critical insights into electron kinetics, ion energy distributions, and reactive species composition, enabling correlations between discharge parameters and surface modification processes. Electrostatic measurements using Langmuir probe diagnostics^32^ reveal that electron populations in such discharges deviate from local thermodynamic equilibrium and often exhibit bi-Maxwellian energy distributions, characterized by a dominant population of low-energy electrons (≈0.2–1.6 eV) coexisting with a high-energy electron tail (≈2.8–8.2 eV). The average electron density, typically on the order of 10^15^ m^–3^, is strongly dependent on operating conditions, increasing with applied power due to electric field enhancement and decreasing with pressure as a result of increased collisional energy losses. Complementary ion mass spectrometry analyses^33,34^ further elucidate molecular fragmentation pathways and the ion energy distribution function (IEDF), which exhibits an inverse dependence on pressure, confirming that higher pressures promote collisional energy dissipation prior to ion–surface interaction. This behavior directly influences surface activation and etching dynamics.^35^

The distinct chemistries of Ar, N_2_, and O_2_ plasmas enable controlled tuning of surface functionalities, following a well-established reactivity trend (O_2_ > N_2_ > Ar), which governs oxidation, nitrogen incorporation, and physical etching mechanisms. These trends are further corroborated by optical emission spectroscopy (OES) studies reported in the literature,^34,35^ indicating that the concentration of excited radicals such as CN, NH, NO, and OH increases with applied power in nitrogen- and oxygen-containing plasmas, reflecting enhanced molecular dissociation and gas-phase reactivity. This established body of knowledge provides a robust framework for interpreting the chemical, morphological, and wettability changes observed in the present work. Such plasma-induced surface modifications have direct implications for composite materials, where improvements in surface energy and interfacial chemistry can enhance fiber–matrix adhesion. The practical benefits of plasma-based surface treatments are evident in their ability to significantly enhance the performance of composite materials. For example, Chahine et al.^36^ observed a 65° decrease in the contact angle of a Polyaryletherketone/carbon fiber (LMPAEK/CF) composite after air plasma treatment, alongside notable improvements in tensile strength (18.75%), flexural strength (8.3%), and Interlaminar Shear Strength (ILSS, 8%). When joining CFRP with dissimilar materials, Shin et al.^37^ reported that low-power oxygen plasma increased the surface energy of an epoxy-based adhesive by 17.7% and improved interlaminar fracture toughness in CFRP-PA6 joints by 51%. Despite these demonstrated benefits, important knowledge gaps remain. Specifically, comparative evaluations of argon, nitrogen, and oxygen low-pressure plasma treatments under identical conditions are still limited in the literature. Additionally, the temporal stability of plasma-induced surface activation, commonly referred to as hydrophobic recovery, remains insufficiently characterized to ensure industrial applicability where delays between treatment and bonding can occur. Moreover, the influence of plasma treatments on the viscoelastic bulk properties of CFRP, such as storage modulus and glass transition temperature, has received comparatively little attention, despite its critical role in assessing potential compromises in composite performance. This study aims to fill these gaps by systematically investigating the effects of Ar, N_2_ and O_2_ plasma treatments at varying powers and exposure times on unidirectional CFRP. The evaluation includes surface chemical analysis (FTIR and XPS), contact angle measurements, which also include the evaluation of hydrophobic recovery time to assess the temporal stability of plasma-induced surface activation, surface morphology characterization through confocal microscopy and SEM, and bulk viscoelastic property assessment by Dynamic Mechanical Analysis (DMA). By integrating surface chemistry, aging behavior, and bulk material property evaluation, this work advances the state of the art and supports the optimization of plasma treatments for structural bonding applications in CFRP composites.

## 2 Materials and Methods

### 2.1 Materials

The manufactured laminates consisted of 36 prepreg layers, each 0.07 mm thick, of the CYCOM 5320-1, supplied by Syensqo. This is a unidirectional carbon fiber/epoxy prepreg system designed for vacuum-bag-only (VBO) or out-of-autoclave (OOA) manufacturing processes.

### 2.2 Laminate and Specimens Manufacturing

A vacuum bag system was used to perform the curing cycle, which consisted of three sequential heating stages: an initial heating to 60 °C for 2 h, followed by heating to 121 °C for an additional 2 h, and a final heating to 177 °C for a further 2 h. No pressure was applied during any stage of the process. The final laminate dimensions were 300 mm × 300 mm × 2.5 mm. Test specimens measuring 50 mm × 10 mm × 2.5 mm were individually sectioned using a TechCut-5 materialographic cutter from Allied High-Tech Products, equipped with a 7″ × 0.025″ × 1/2″ diamond disc to achieve a superior edge finish and minimize delamination. Before plasma treatment, surface decontamination of the samples was conducted via an ultrasonic bath in distilled water at room temperature for 30 min, followed by cleaning with isopropyl alcohol and drying in an oven at 40 °C for 24 h.

### 2.3 Low-Temperature Plasma Treatment

Low-temperature plasma treatments were performed using three different gases (argon, nitrogen, and oxygen) under controlled conditions: power levels of 20 and 50 W, a fixed pressure of 100 mTorr, and treatment times of 600, 1200, and 1800 s. Prior to the introduction of the process gas, the reactor was evacuated to a pressure of approximately 1 mTorr to minimize the presence of residual oxygen. This initial vacuum step further reduces the likelihood of contamination from atmospheric oxygen during the plasma treatment. Figure 1a provides a schematic representation of the reactor system where the main components are the vacuum pumps (labeled as 1 and 2), the radio frequency (RF) power source (3), the gas flow control valves (4), and the reactor chamber (5). Figure 1b offers an internal view of the reactor, highlighting the gas inlet (1), the sample holder (2), and the gas outlet (3). The gas flows internally from the inlet (1) toward the outlet (3), establishing a diagonal path across the sample surface. It should be noted that all subsequent surface characterization analyses were conducted on the upper surface of the samples, which was directly exposed to the plasma. The lower surface, in contact with the bottom electrode, was shielded from direct plasma interaction and therefore did not undergo the same degree of modification. As a result, the treatment selectively modifies only the plasma-exposed side of the material.

**Figure 1:**
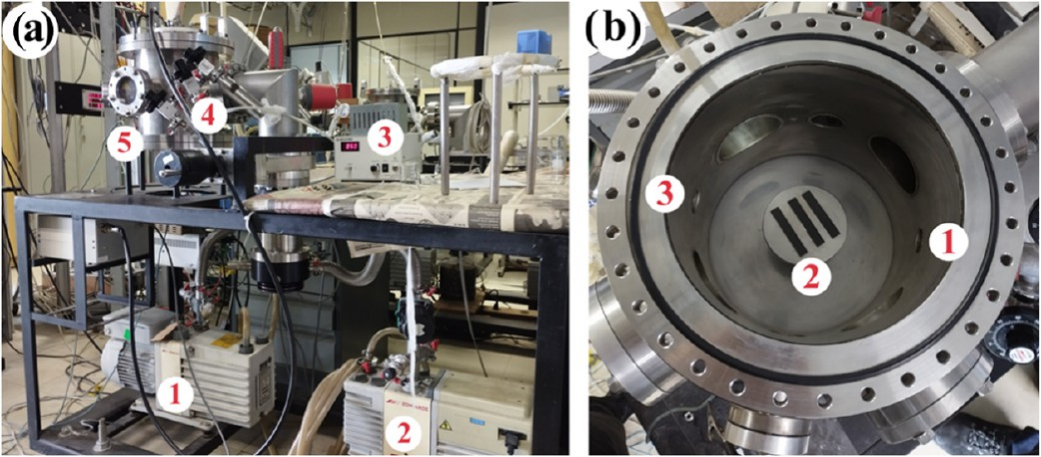
(a) Reactor system overview; (b) Inside view of the reactor.

The reactor was operated with argon, nitrogen, and oxygen gases, as shown respectively in Figure 2a,b,c. The distinct colors visible in these images arise from the excitation of gas atoms and their characteristic emission of light at specific wavelengths when electrically energized, visually illustrating this fundamental phenomenon.

**Figure 2:**
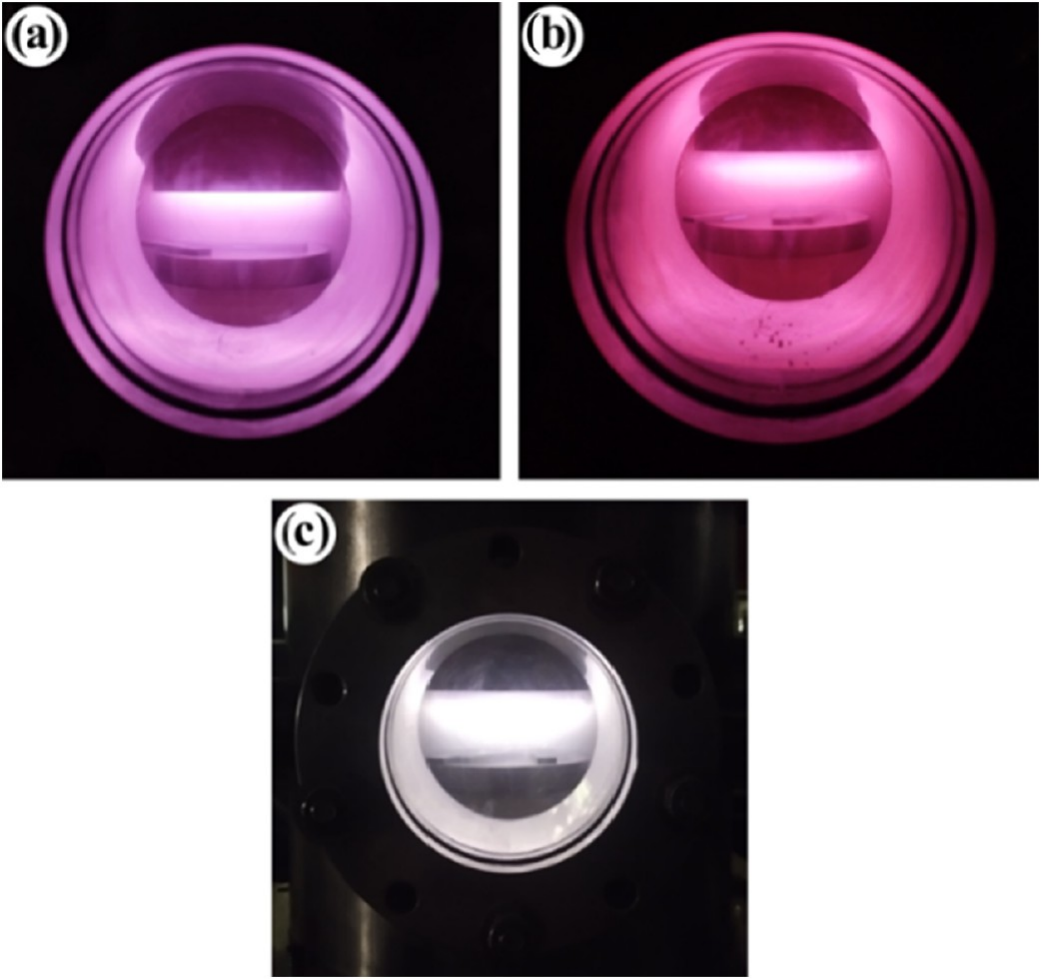
Reactor operating with (a) Argon; (b) Nitrogen; (c) Oxygen.

### 2.4 Roughness Analysis in Confocal Microscope

To assess the topographical differences between untreated and plasma-treated surfaces, three-dimensional surface analyses were performed using a LEICA DCM 3D optical metrology system, equipped with a 10× confocal objective and a 405 nm blue LED light source. Ten measurements were acquired from distinct regions measuring 1.27 × 0.95 mm^2^ for each treatment condition. The analyzed surface parameters included the arithmetic mean roughness (Sa), root-mean-square roughness (Sq), maximum peak height (Sp), maximum pit depth (Sv), and maximum height (Sz).

### 2.5 Contact Angle

Contact angle measurements were performed using a Ramè-Hart 100-00 goniometer with the sessile drop method. The contact angle values were determined as the arithmetic mean of 10 measurements taken at evenly distributed points across the entire surface of each sample. For the analysis, 100 μL droplets of deionized water were dispensed at room temperature.

### 2.6 Fourier-Transform Infrared (FTIR) Spectroscopy

Fourier transform infrared (FTIR) spectroscopy in attenuated total reflectance mode (FTIR-ATR) was employed to investigate the impact of plasma treatments on the functional groups present in the samples. Spectra were acquired using a PerkinElmer Spectrum GX FTIR spectrophotometer equipped with an ATR accessory, spanning a wavenumber range of 4000–400 cm^–1^, with a spectral resolution of 4 cm^–1^ and 64 coadded scans. All measurements were performed in ATR mode, and data are presented in transmittance units for consistency in data analysis. To facilitate visual comparison and highlight possible changes in functional groups, each spectrum was normalized by its maximum absorbance, ensuring a maximum intensity of 1. This normalization does not affect the band positions or relative shapes and allows clearer overlay and comparison of the spectra.

### 2.7 X-ray Photoelectron Spectroscopy (XPS)

X-ray Photoelectron Spectroscopy (XPS) analyses were conducted using a Thermo Scientific K-Alpha spectrometer equipped with a monochromatic Al Kα radiation source (photon energy = 1486 eV) to investigate the chemical composition and surface functionalization of the CFRP before and after plasma treatment. Survey spectra were acquired to identify the main constituent elements (C, O, and N), and their atomic concentrations were calculated from the integrated peak areas of the corresponding photoelectron lines, normalized to the total spectral area. High-resolution spectra of the C 1s, O 1s, and N 1s regions were recorded to enable precise peak deconvolution and identification of specific chemical states. The relative abundance of each functional group was determined from the fractional contribution of its fitted component to the total peak area within the corresponding spectrum. All spectral fitting and quantitative analyses were performed using CasaXPS software.

### 2.8 Surface Characterization Using Scanning Electron Microscopy (SEM)

A Zeiss EVO LS15 scanning electron microscope, operating in low-voltage mode (LV-SEM), was employed to analyze microscopic alterations on the sample surface induced by plasma treatments. Prior to imaging, the samples were coated with a gold layer to improve surface conductivity. The coating procedure resulted in an approximate gold thickness of 24.40 nm.

### 2.9 Dynamic-Mechanical Analysis (DMA)

Dynamic mechanical analysis (DMA) was conducted to assess potential alterations in the viscoelastic and thermal properties of the samples induced by plasma treatments. The analyses were performed using a Thermal Analyzer SII Exstar 6000 (model DMS 6100) operating in dual cantilever mode. Experimental conditions were as follows: temperature range of 25–260 °C, heating rate of 3 °C/min, oscillation frequency of 1 Hz, and displacement amplitude of 10 μm.

## 3 Results and Discussion

### 3.1 Mechanical Surface Activation Analysis

The results of the surface roughness analyses, as well as the percentage differences relative to the control sample, are presented in Tables 1 and 2, respectively. As shown in Table 1, the treatment using argon gas did not result in significant changes in the surface roughness of the treated samples, except under the condition of the lowest power (20 W) and shortest treatment duration (600 s). In this specific case, an increase of approximately 15–20% was observed, particularly in the maximum valley depth, suggesting a mild surface ablation process induced by the cathodic sputtering action of argon ions.^38^ This effect was not pronounced under longer plasma exposure times or higher power levels. Three-dimensional surface reconstruction via confocal microscopy (Figure 3) corroborates these findings, showing no significant differences between the control surface (Figure 3a) and the surface treated with argon plasma (Figure 3b). This indicates that argon gas, when used as the plasma activation medium, was not effective in promoting mechanical activation of the surface, even at longer treatment times or higher power levels. It is important to note that due to the inherent characteristics of plasma treatment, surface modifications occur locally and are influenced by several factors such as the random impact locations of energized atoms and the heterogeneity of plasma exposure across the sample surface, resulting in variations within the measured roughness values. Furthermore, while the current analysis focuses on surface roughness at the microscopic scale, plasma treatment also induces nanoscale roughening of the polymeric matrix in CFRP surfaces, which can significantly influence wetting behavior and mechanical interlocking at the adhesive interface.^36^ Such nanoscale modifications, although beyond the resolution of the present confocal microscopy measurements, likely contribute to the observed improvements in wettability and adhesion performance.

**Figure 3:**
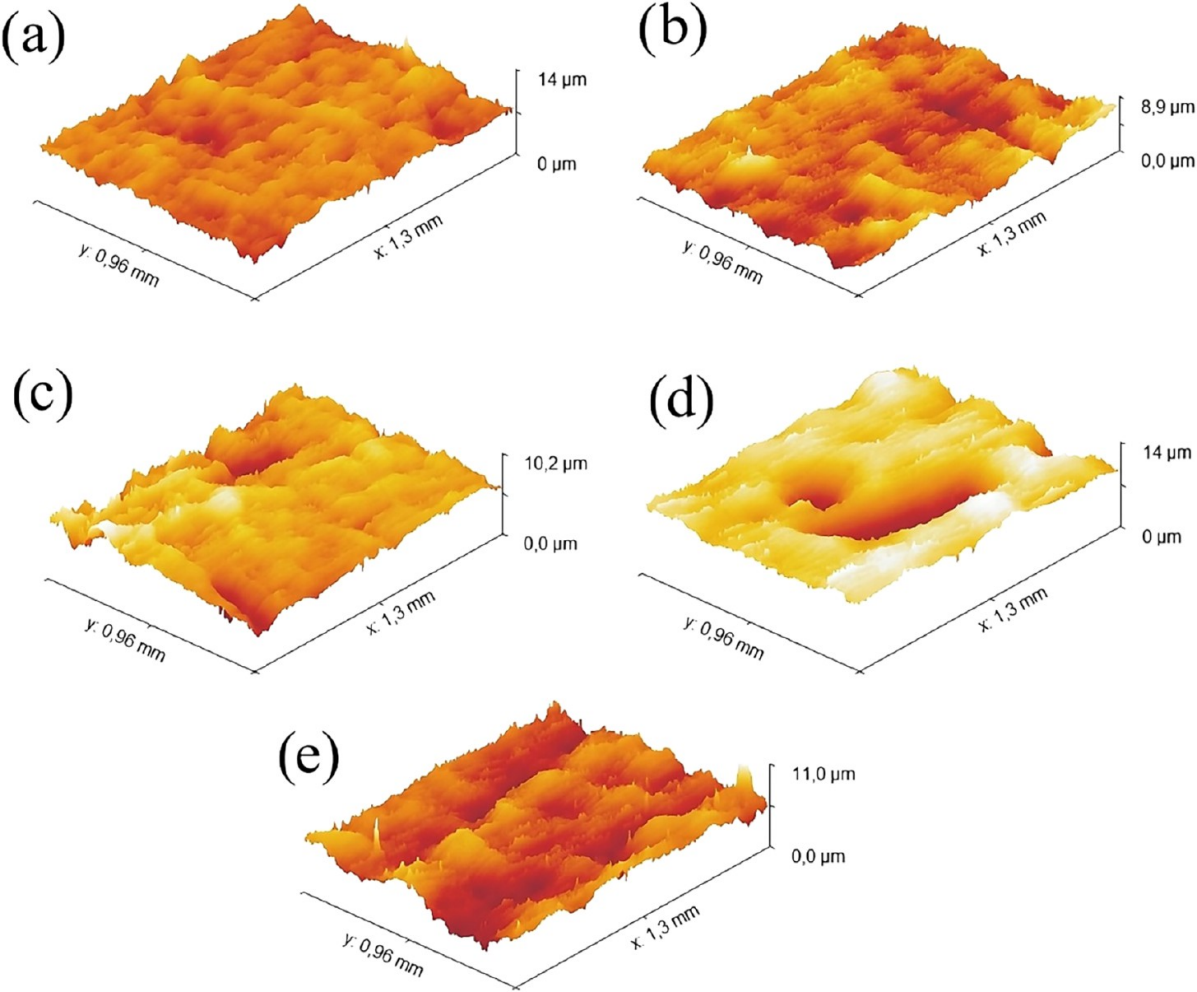
3D reconstructions by confocal microscopy of the samples: (a) Control; (b) Argon–20 W–600 s; (c) Nitrogen–20 W–1200 s; (d) Nitrogen–20 W–1800 s; and (e) Oxygen–20 W–600 s.

**Table 1:** Summary of the Roughness Parameters Measured after Plasma Treatments

			roughness parameters average (μm)				
gas	power (W)	time (s)	Sa	Sq	Sp	Sv	Sz
Ar	20	600	1.26 ± 0.14	1.53 ± 0.15	5.34 ± 1.44	8.08 ± 3.55	13.42 ± 3.76
		1200	1.01 ± 0.39	1.09 ± 0.66	3.95 ± 0.99	5.97 ± 0.76	9.92 ± 1.39
		1800	1.00 ± 0.21	1.23 ± 0.23	4.41 ± 0.93	5.24 ± 0.62	9.65 ± 1.21
	50	600	1.18 ± 0.09	1.44 ± 0.10	4.86 ± 0.73	5.79 ± 0.56	10.65 ± 0.97
		1200	1.11 ± 0.19	1.35 ± 0.20	4.27 ± 0.53	7.66 ± 3.68	11.93 ± 3.93
		1800	1.24 ± 0.36	1.53 ± 0.47	4.59 ± 0.83	7.16 ± 5.07	11.75 ± 5.43
N2	20	600	1.60 ± 0.24	1.95 ± 0.28	5.58 ± 1.50	6.15 ± 0.81	11.73 ± 2.18
		1200	2.58 ± 0.15	3.03 ± 0.18	8.96 ± 2.64	8.09 ± 1.72	17.04 ± 2.63
		1800	1.88 ± 0.33	2.31 ± 0.38	8.91 ± 9.35	14.05 ± 13.86	22.97 ± 15.49
	50	600	1.28 ± 0.20	1.56 ± 0.22	4.70 ± 0.52	6.25 ± 0.90	10.95 ± 0.98
		1200	1.90 ± 0.18	2.37 ± 0.24	7.45 ± 2.74	12.14 ± 3.57	19.59 ± 5.33
		1800	1.35 ± 0.30	1.98 ± 0.48	8.86 ± 7.53	13.13 ± 6.10	21.99 ± 13.22
O2	20	600	3.21 ± 0.12	3.75 ± 0.14	9.72 ± 1.79	9.33 ± 1.59	19.05 ± 1.94
		1200	2.43 ± 0.20	2.88 ± 0.25	7.52 ± 0.70	8.27 ± 1.31	15.79 ± 1.60
		1800	1.70 ± 0.16	2.04 ± 0.20	8.18 ± 3.32	8.53 ± 1.43	16.71 ± 3.68
	50	600	1.72 ± 0.14	2.03 ± 0.16	6.01 ± 1.23	7.21 ± 1.18	13.22 ± 1.73
		1200	1.53 ± 0.41	1.84 ± 0.49	7.59 ± 2.38	6.04 ± 0.67	13.63 ± 2.46
		1800	1.34 ± 0.08	1.61 ± 0.10	7.00 ± 2.39	6.02 ± 0.92	13.02 ± 2.55

**Table 2:** Range of Difference Versus Control Sample

			range of difference versus control (%)				
gas	power (W)	time (s)	Sa	Sq	Sp	Sv	Sz
Ar	20	600	16.7	14.3	–1.7	23.0	11.8
		1200	–6.0	–18.7	–27.3	–9.2	–17.4
		1800	–7.4	–7.9	–18.9	–20.2	–19.6
	50	600	9.1	7.3	–10.6	–11.8	–11.2
		1200	2.7	1.2	–21.4	16.6	–0.6
		1800	14.9	14.6	–15.5	9.0	–2.1
N_2_	20	600	48.5	45.9	2.7	–6.4	–2.3
		1200	138.5	126.7	64.8	23.1	42.0
		1800	73.8	72.8	64.0	114.0	91.4
	50	600	18.5	16.3	–13.5	–4.9	–8.8
		1200	75.5	77.0	37.1	84.8	63.2
		1800	25.3	47.9	63.0	99.9	83.2
O_2_	20	600	197.6	180.2	79.0	42.1	58.8
		1200	125.0	115.2	38.3	25.9	31.5
		1800	57.5	52.2	50.6	29.8	39.2
	50	600	59.1	52.0	10.7	9.7	10.2
		1200	41.8	37.4	39.7	–8.1	13.6
		1800	24.3	20.3	28.8	–8.3	8.5

In contrast, nitrogen plasma treatment led to more pronounced surface modifications under specific conditions. Notably, at 20 W with treatment durations of 1200 and 1800 s, significant increases in surface roughness parameters were observed. For the 1200 s condition, the average roughness increased by approximately 140%, while the 1800 s condition resulted in a 115% increase in maximum valley depth. Figure 3c,d illustrate the emergence of deeper valley regions in samples treated with nitrogen plasma for 1200 and 1800 s, respectively, in comparison to the control sample (Figure 3a). These cavities allow structural adhesives to penetrate surface irregularities via capillary action, increasing the contact area between the adherend surface and the adhesive. This facilitates mechanical interlocking, thereby enabling improvements in mechanical properties. Under higher power and longer treatment durations, the changes in surface roughness were smaller or comparable to those obtained at lower power and shorter exposure times. This nonlinear behavior is attributed to saturation effects and possible surface degradation under more intense plasma conditions, indicating that prolonged or high-power treatments do not necessarily lead to greater surface activation. Consequently, milder plasma conditions can be more effective for this composite type, with the added advantages of reduced energy consumption and higher throughput. The abstraction of atoms from the polymeric network that exhibit high chemical affinity with nitrogen, in conjunction with the high electronegativity of nitrogen (3.04), is proposed as a mechanism for the formation of active sites, thereby contributing to increased surface roughness.^39^ Observations by Esposito et al.^40^ support this hypothesis, as plasma-treated poly­(lactic-*co*-glycolic acid) surfaces exhibited rough morphologies and deep cavities.

A similar trend was observed for oxygen plasma treatment, where the highest efficiency in roughness enhancement was achieved at the lowest reactor power (20 W) and shortest treatment time (600 s). Under this condition, the average roughness of the composite increased by nearly 200%. Figure 3e highlights the increase in average surface roughness of the treated material compared to the control surface. A significant rise in surface irregularities can be observed, which favors mechanical interlocking following the application of a structural adhesive. At longer treatment durations and higher power levels, the changes were either comparable or less effective, indicating that for this unidirectional composite, oxygen plasma treatment is also more efficient for mechanical surface activation under reduced exposure times and low reactor power. This operational window is advantageous for minimizing energy consumption and maximizing productivity. The underlying mechanism appears to parallel what was observed with nitrogen plasma. Elements within the polymer matrix exhibiting strong chemical affinity with oxygen, together with oxygen’s high electronegativity (3.44) and valence electron configuration, promote the formation of oxygen-containing functional groups (e.g., hydroxyl, carbonyl, carboxyl) on the surface. These groups act as chemically reactive sites (“active sites”), increasing surface polarity and energy, which facilitate enhanced interfacial interactions and adhesion. These chemical modifications promote localized microetching and subtle surface irregularities at the micrometer scale, leading to a modest increase in surface roughness. Such changes enhance mechanical interlocking, particularly relevant for structural adhesive applications, by improving capillary action and surface adhesion.^39–41^

### 3.2 Surface Wettability and Hydrophobic Recovery

Table 3 presents the contact angle measurements following each plasma treatment, alongside the percentage differences relative to the control sample. Notably, argon plasma treatment resulted in significant reductions in contact angle, despite minimal alterations in surface roughness. Specifically, treatments at 20 and 50 W led to decreases of approximately 90 and 95%, respectively. This enhancement in wettability is attributed to the incorporation of oxygen-containing functional groups, as reported by Luque-Agudo et al.^42^ and Gustus & Wegewitz,^43^ which increase surface hydrophilicity. Although argon itself does not contain oxygen, the plasma treatment activates the surface by generating reactive sites that readily react with atmospheric oxygen upon exposure to ambient air, leading to the indirect formation of these oxygen-containing groups. The observed effect is likely due to the argon plasma’s n capability, effectively removing surface contaminants that form weak boundary layers, thereby exposing a more reactive and hydrophilic surface enriched with oxygen functionalities.

**Table 3:** Contact Angles of Water on Plasma-Treated Samples^a^

gas	power (W)	time (s)	contact angle (deg)	range of difference versus control (%)
Ar	20	600	18.8 ± 11.1	76.1
		1200	14.8 ± 6.9	81.1
		1800	6.7 ± 2.1	91.5
	50	600	5.7 ± 1.3	92.8
		1200	4.2 ± 1.2	94.7
		1800	7.4 ± 3.4	90.6
N_2_	20	600	11.5 ± 1.5	85.3
		1200	N.M.	
		1800	N.M.	
	50	600	N.M.	
		1200	N.M.	
		1800	N.M.	
O_2_	20	600	12.2 ± 1.5	84.5
		1200	10.2 ± 1.7	87.0
		1800	5.9 ± 1.1	92.5
	50	600	5.7 ± 1.54	92.7
		1200	4.5 ± 0.89	94.3
		1800	11.5 ± 3.43	85.3

a N.M. = Not Measurable.

In contrast, nitrogen plasma treatment exhibited an even more pronounced effect on surface wettability. A measurable contact angle was only obtainable under conditions of 20 W power and 600 s exposure, showing an increase of approximately 85%. For longer treatment durations and higher power settings, precise contact angle measurements were unattainable due to the surfaces achieving superhydrophilic states. This phenomenon can be explained by the formation of deep valleys observed in roughness measurements after 1200 s of treatment at 20 W, coupled with the introduction of nitrogen-containing functional groups such as amines (−NH_2_), imines (=NH), nitriles (–C≡N), and amides (−CONH_2_). These groups exhibit a strong chemical affinity for water molecules. Similar to argon plasma, the nitrogen plasma activates the surface chemically, promoting indirect oxidation by atmospheric oxygen that results in the formation of oxygen-containing functional groups. Additionally, the generation of high-energy species like ions, electrons, and nitrogen radicals during plasma treatment creates active sites, significantly enhancing surface reactivity.^44–46^

Oxygen plasma treatment also induced substantial changes in the composite’s wettability. A treatment duration of 600 s at 20 W resulted in an approximate 85% increase in hydrophilicity, with longer exposures yielding increases up to 93%. Similar outcomes were observed at higher power levels. Unlike nitrogen plasma, which formed deep cavities, oxygen plasma uniformly increased surface roughness. This mechanical activation, combined with chemical activation through the incorporation of oxygen-containing groups (e.g., hydroxyls, carbonyls, carboxyls), the creation of active sites, and the generation of high-energy species, collectively enhances surface reactivity and, consequently, wettability.^47,48^

Figure 4 illustrates the contact angle variations of treated samples as a function of plasma exposure time. It is noteworthy that increasing the power from 20 to 50 W did not yield significant improvements, indicating that lower power settings are sufficient for effective mechanical and potential chemical activation of the composite surface. Similarly, optimal treatment durations were identified: 600 s for argon and oxygen plasmas, and 1200 to 1800 s for nitrogen plasma. For applications involving structural adhesives, rapid, clean, and cost-effective surface treatments are crucial for both mechanical property enhancements and productivity gains.

**Figure 4:**
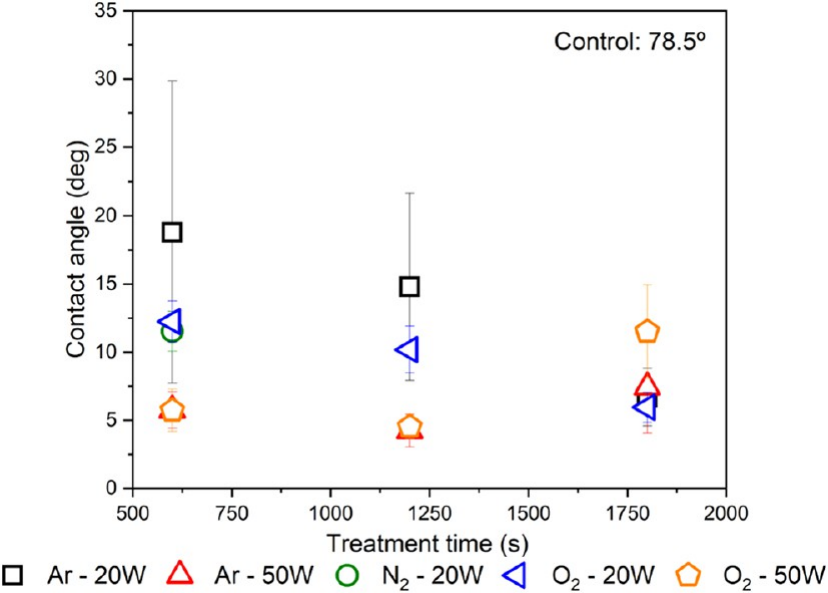
Contact angle as a function of treatment time for the five most effective parameter combinations in terms of inducing significant modifications to the adherend surface.

To evaluate the durability of plasma-induced surface activation, a 70-h aging study was conducted using samples that exhibited the most pronounced increases in surface roughness, as determined by comprehensive confocal microscopy analyses of all treatment conditions, as shown in Figure 5. The same sample from each treatment condition was used for contact angle measurements over time post-treatment to ensure consistency and minimize variability related to sample differences. Samples were exposed to ambient air, replicating realistic industrial environments. This approach allows for a thorough evaluation of plasma-treated surfaces’ stability and interaction with atmospheric conditions, thereby providing relevant insights into their practical applications. Contact angle measurements were taken at regular intervals until stabilization was observed. The highest plasma efficiency was recorded immediately post-treatment, before any significant atmospheric interactions. After 1 h, argon-treated composites showed a 31% increase in contact angle; nitrogen-treated samples (1200 and 1800 s) exhibited an absolute increase of approximately 22°, and oxygen-treated samples showed an increase of about 23°. Extending the post-treatment period to 6 h, the contact angle increases were approximately 92% for argon, 36° for nitrogen (1200 s), 28° for nitrogen (1800 s), and 37° for oxygen. After 12 h, nitrogen-treated surfaces began to exhibit more pronounced increases in contact angle, stabilizing at approximately 61° (1200 s) and 62° (1800 s). This trend is attributed to the high reactivity of plasma-generated species, active sites, and high-energy entities with atmospheric moisture and contaminants, leading to increased contact angles.^46–48^ Argon-treated surfaces stabilized around 54°, while oxygen-treated surfaces maintained the lowest stabilized contact angle (∼42°) and exhibited the least variation over time.

**Figure 5:**
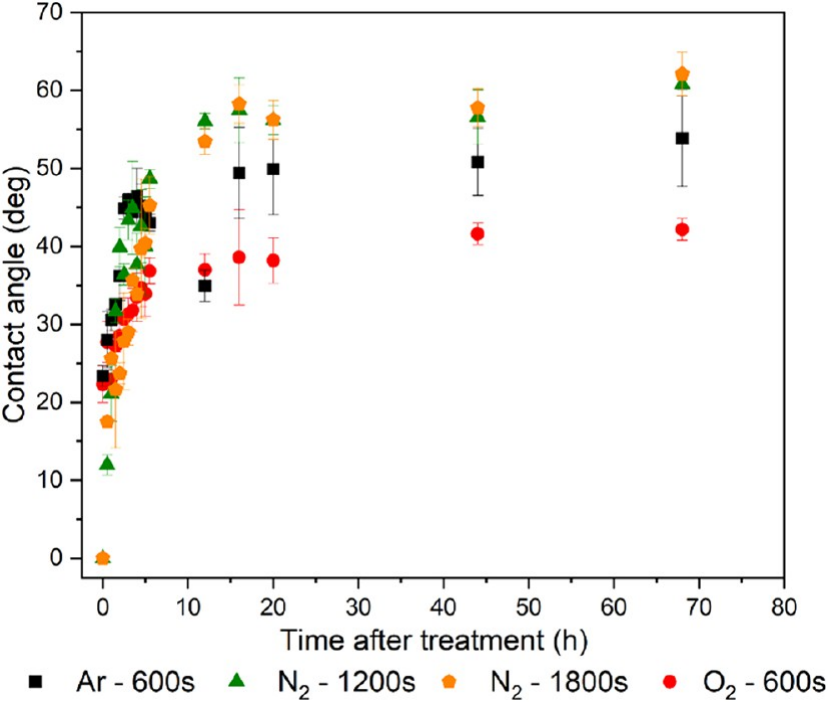
Increase in contact angle over time following surface treatment.

Despite the similarities in factors enhancing wettability for nitrogen and oxygen plasma treatments, differences in hydrophobic recovery rates and stabilized contact angles can be ascribed to the nature of the functional groups and molecular rearrangements. Oxygen-derived functional groups such as hydroxyls (−OH), carbonyls (C=O), and carboxyls (−COOH), are highly polar and form stable hydrogen bonds with environmental moisture, ensuring long-term stability. Conversely, nitrogen-derived groups such as amines (−NH_2_), imines (=NH), nitriles (–C≡N), and amides (−CONH_2_), while also hydrophilic, are less so than their oxygen counterparts, resulting in reduced stability and accelerated hydrophobic recovery.^49–51^ Postplasma treatment, the energized surface rich in polar groups undergoes molecular reorientation over time due to environmental interactions. Additionally, nonpolar groups from the original epoxy matrix may migrate to the surface. These phenomena are more pronounced with nitrogen plasma treatments due to the lower stability of nitrogen-containing groups compared to oxygen-containing ones.^52^ It is important to emphasize that the primary objective of this aging study was not to indefinitely suppress hydrophobic recovery, but rather to establish the operational time frame within which the surface remains sufficiently activated for practical applications, particularly structural bonding. The results indicate that, under all treatment conditions investigated, the surfaces preserved a high degree of activation for a period of at least 10 h, which aligns with typical industrial processing intervals between plasma treatment and adhesive bonding. Consequently, although a gradual reduction in surface activation is observed over extended periods, the treatment remains highly applicable and effective in scenarios where post-treatment operations are carried out within this defined time frame.

### 3.3 Spectroscopic Characterization of Chemically Activated Surfaces: FTIR and XPS

Figure 6 shows the normalized FTIR spectra of the samples that exhibited the most pronounced surface modifications, as determined by confocal microscopy roughness analysis. The observed absorption bands are consistent with those typically found in epoxy-based materials, as reported in studies.^53,54^ Comparison of the spectra reveals no substantial changes in the position or intensity of the pre-existing absorption bands, nor the emergence of new peaks that would indicate the formation of new functional groups or chemical rearrangements resulting from plasma treatment. This suggests that the plasma processes did not significantly alter the bulk chemical structure of the epoxy matrix, or that any changes induced remained below the detection limit of the FTIR technique. To more accurately assess potential chemical modifications, in-depth XPS analyses were performed.

**Figure 6:**
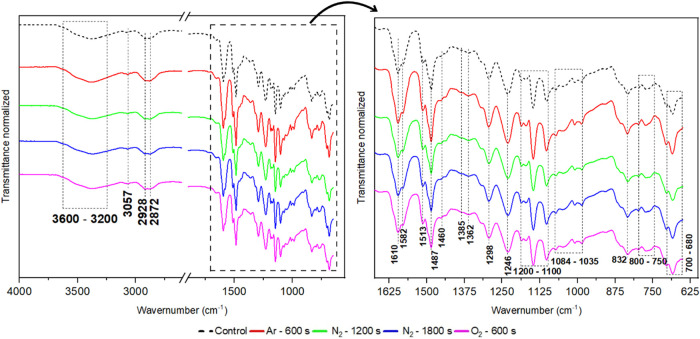
Fourier-transform infrared (FTIR) spectra of plasma-treated samples that exhibited the most significant increases in surface roughness.

Figure 7 presents the full XPS spectra for the samples that exhibited the most significant surface modifications, as previously identified by confocal microscopy roughness analysis. These spectra reveal the presence of C 1s, O 1s, N 1s, and Si 2s/Si 2p species. However, the silicon peaks (Si 2s and Si 2p) were disregarded as they are not the focus of this analysis. It is important to note that all XPS elemental compositions discussed here are expressed as relative atomic percentages normalized to the total detected elements. Therefore, an apparent increase in the proportion of a given element or functional group does not necessarily indicate an absolute increase in its surface amount, but rather a change in its relative contribution due to variations in other elements present. In the control sample (Figure 7a), a distinct F 1s peak at 689.08 eV indicates the presence of fluorine-containing compounds.^55^ Fluorine is not expected in the epoxy matrix, suggesting contamination from fluorinated mold release agents, handling processes, or residuals from prepreg manufacturing. Notably, after plasma treatments, argon (20 W, 600 s; Figure 7b), nitrogen (20 W, 1200 and 1800 s; Figure 7c,d), and oxygen (20 W, 600 s; Figure 7e), the F 1s peak is absent. This confirms that plasma treatment not only effectively cleans and decontaminates the surface, but also that any fluorine present was likely superficial and not chemically bonded to the composite matrix. Therefore, plasma processes proved highly efficient in both surface cleaning and chemical activation.

**Figure 7:**
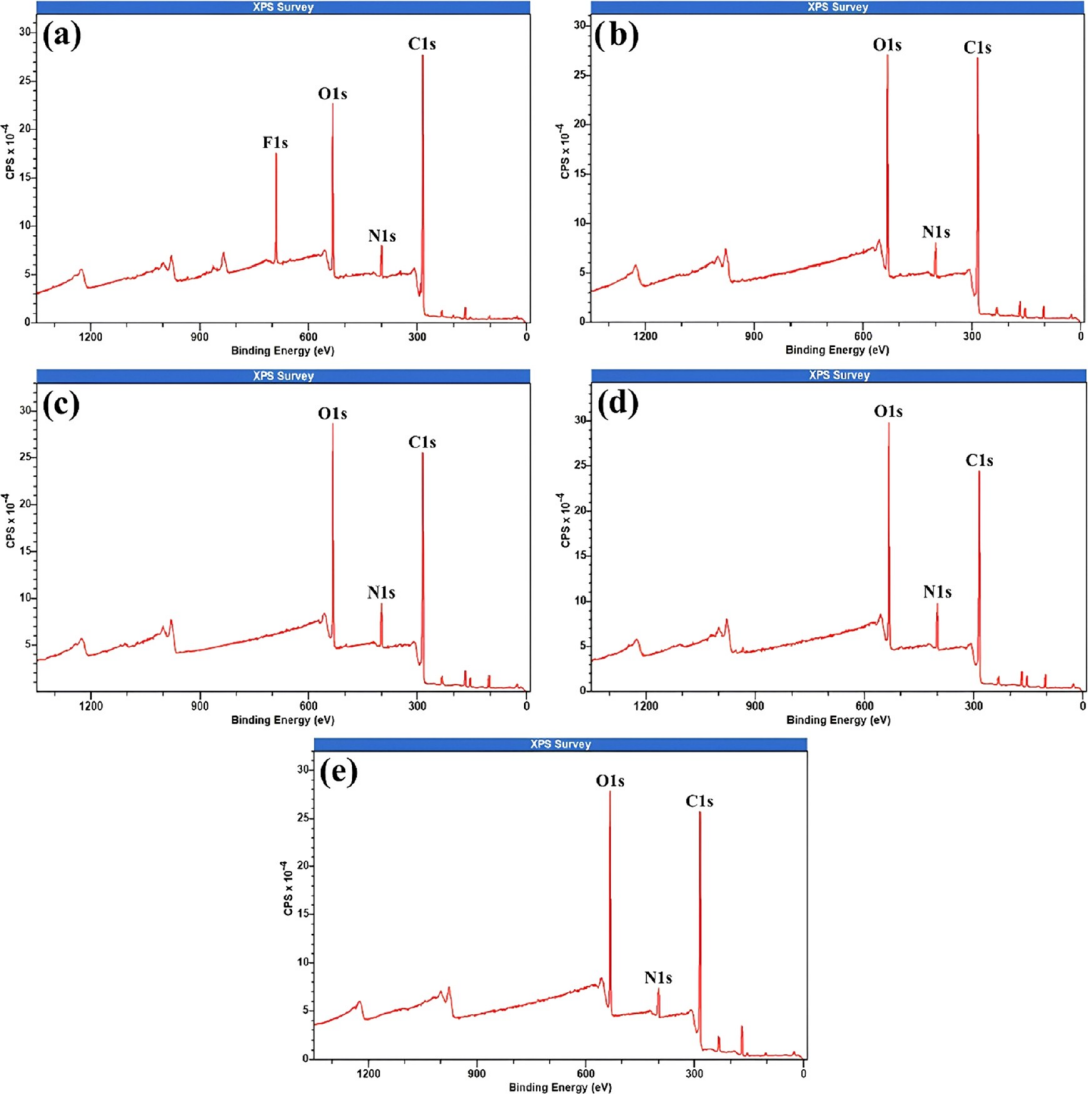
X-ray Photoelectron Spectroscopy (XPS) of samples (a) Control; (b) Argon–20 W–600 s; (c) Nitrogen–20 W–1200 s; (d) Nitrogen–20 W–1800 s; and (e) Oxygen–20 W–600 s.

Table 4 summarizes the elemental composition of the composite surface before and after plasma treatment. Considering the relative nature of XPS data, In addition to the removal of approximately 20% of surface contaminants, plasma treatments resulted in a higher relative proportion (16–18%) in oxygen and oxygenated functional groups, suggesting enhanced the surface reactivity.^56^ A nitrogen concentration increase, ranging from 1.3 to 3.7%, was also observed, contributing further to surface chemical activation. Regarding carbon content, only the argon plasma treatment showed a noticeable increase (∼3%), which is consistent with its tendency to promote rearrangement of pre-existing functional groups rather than the incorporation of new ones. For nitrogen and oxygen plasmas, changes in carbon content were minimal, except for nitrogen plasma at 1800 s, where a slight decrease (∼1.7%) may be related to surface saturation or etching effects under prolonged exposure. This behavior is coherent with the different mechanisms of each gas and the balance between incorporation of new groups and modification of existing ones. Pizzorni et al.^57^ demonstrated that introducing functional groups via plasma treatments can improve the performance of adhesively bonded CFRP joints by up to 40%. Such chemical modifications, combined with effective surface cleaning, highlight the dual role of plasma treatment in simultaneously enabling chemical activation and decontamination, thereby optimizing surfaces for high-performance bonding applications.

**Table 4:** Summary of Surface Chemical Composition (in Atomic%) for Control and Plasma-treated Samples^a^

	elemental composition (%)			
sample	carbon	nitrogen	oxygen	fluorine
control	44.00	5.22	30.73	20.05
Ar_20 W_600 s	47.10	6.54	46.35	N.D.
N_2__20 W_1200 s	44.24	8.46	47.30	N.D.
N_2__20 W_1800 s	42.28	8.90	48.82	N.D.
O_2__20 W_600 s	44.24	7.59	48.17	N.D.

a N.D. = Not Detected.

To better understand changes in surface chemistry after each plasma treatment, high-resolution spectra for C 1s, N 1s, and O 1s are shown in Figures 8, 9, and 10, respectively. In the C 1s spectra (Figure 8), the control sample (Figure 8a) shows three peaks: C–C at 284.49 eV (66.45%), C–N at 285.84 eV (30.19%), and another at 287.94 eV related to carboxylic acids (O–C=O) (3.37%). After argon plasma treatment (20 W, 600 s; Figure 8b), there is a slight increase in the C–C peak (around 1.8%) and in the carboxylic acids group (0.8%), while a sharp decrease in the C–N peak (15.6%) is observed. One new peak appears at 286.26 eV, corresponding to carbonyl (C=O) or ether (O–C–O) groups (12.94%). The argon plasma’s mild sputtering effect removes weakly bound surface contaminants and may induce slight surface ablation, creating reactive sites. In contrast, for O_2_ and N_2_ plasmas, surface cleaning is primarily driven by oxidative etching and desorption of loosely bound contaminants, rather than by physical sputtering. The reactive sites generated, particularly in Ar plasma, rapidly interact with air, forming oxygen-containing groups such as carbonyls, ethers, and carboxylic acids.^58^ The increase in C–C bonds may result from removing surface oxygen and hydrogen atoms, causing molecular rearrangement and forming more stable carbonaceous structures.^59^

**Figure 8:**
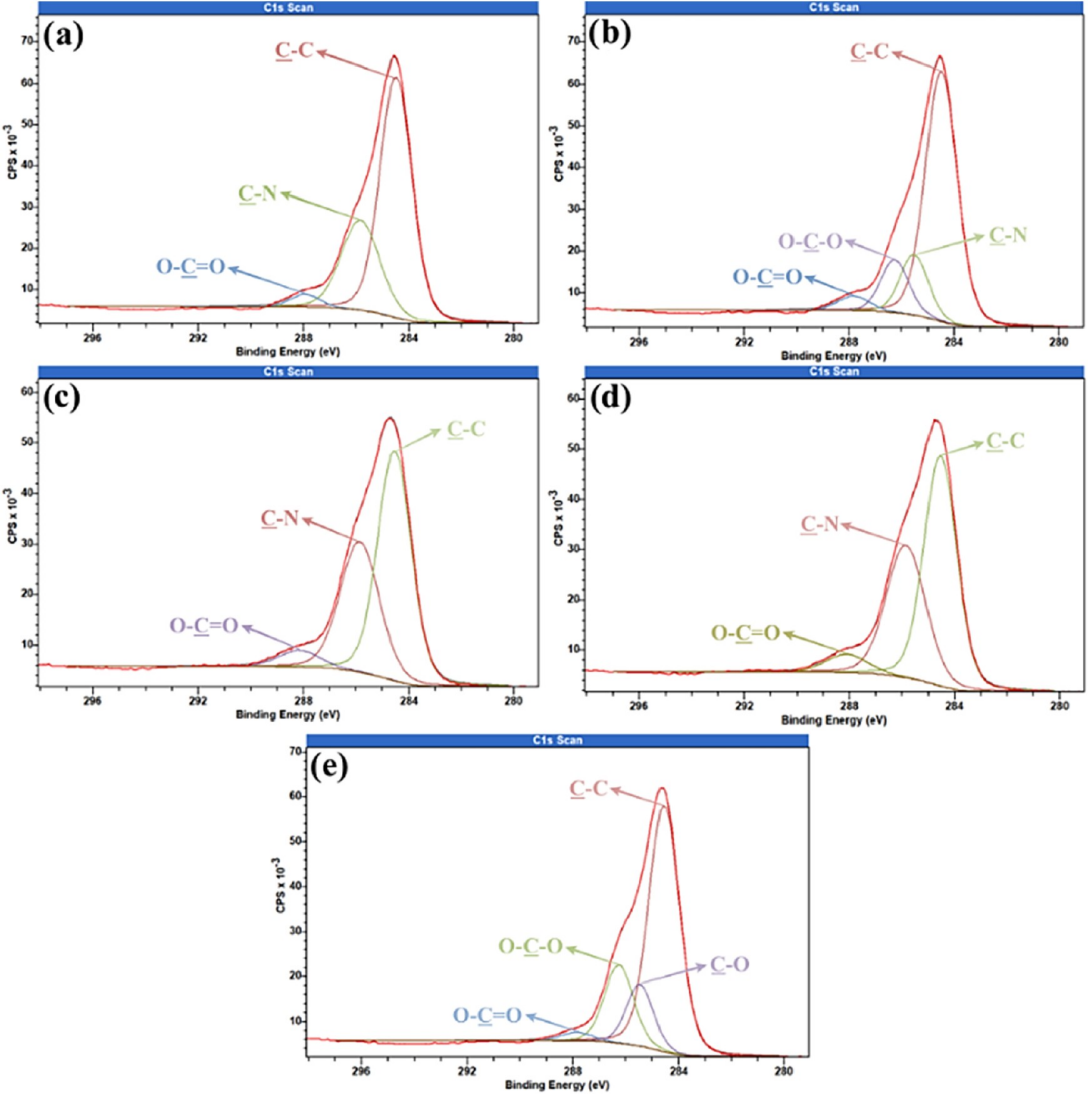
High resolution C 1s of samples (a) Control; (b) Argon–20 W–600 s; (c) Nitrogen–20 W–1200 s; (d) Nitrogen–20 W–1800 s; and (e) Oxygen–20 W–600 s.

**Figure 9:**
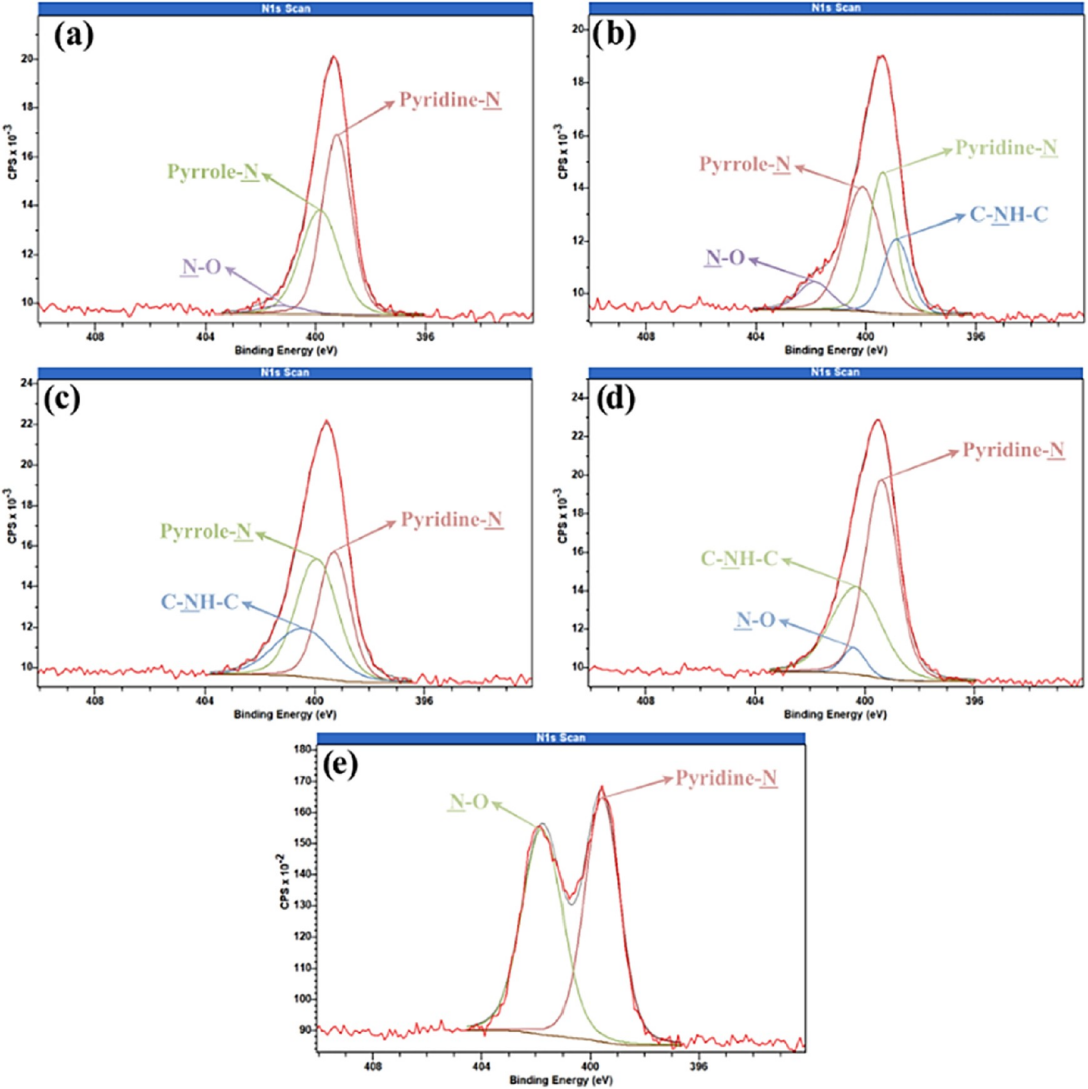
High resolution N 1s of samples (a) Control; (b) Argon–20 W–600 s; (c) Nitrogen–20 W–1200 s; (d) Nitrogen–20 W–1800 s; and (e) Oxygen–20 W–600 s.

**Figure 10:**
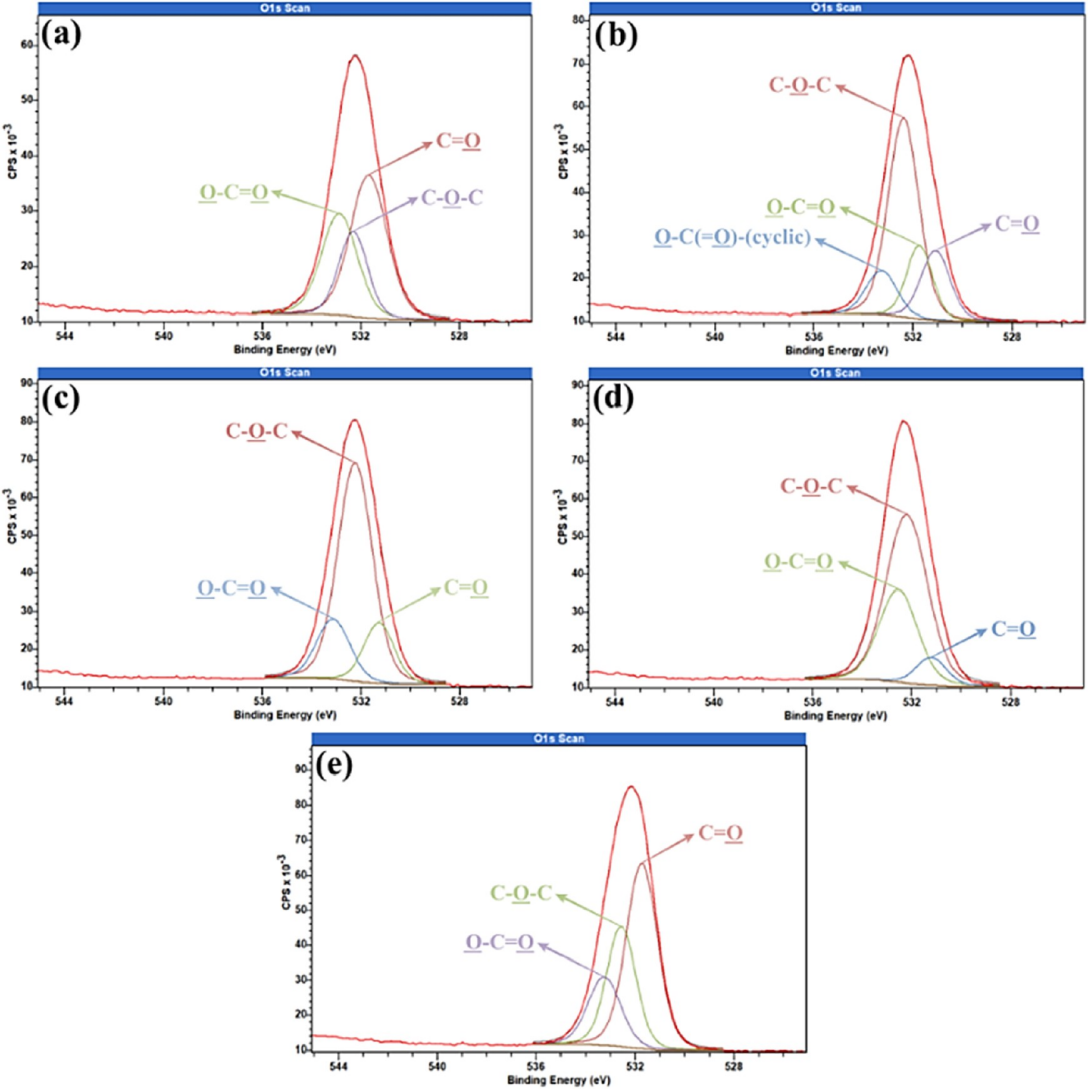
High resolution O 1s of samples (a) Control; (b) Argon–20 W–600 s; (c) Nitrogen–20 W–1200 s; (d) Nitrogen–20 W–1800 s; and (e) Oxygen–20 W–600 s.

For nitrogen plasma at 1200 s (Figure 8c), the original peaks persist but with shifted concentrations: C–C at 284.55 eV (56.88%) and C–N at 285.86 eV (37.91%). This suggests a ∼9.6% reduction in graphitic carbon and a corresponding increase in nitrogen functionality. Extending the treatment to 1800 s (Figure 8d) intensifies this trend, with C–N bonds rising slightly to 38.49% and C–C dropping marginally to 55.72%, indicating that the surface is approaching a saturation point in nitrogen functionalization. The modest changes between 1200 and 1800 s imply that increasing exposure time beyond 1200 s yields diminishing returns in surface modification, likely due to the exhaustion of reactive sites and stabilization of formed functional groups. Additionally, longer plasma exposures may risk surface degradation, emphasizing the need to balance treatment duration for effective functionalization without compromising material integrity.

Oxygen plasma treatment (20 W, 600 s; Figure 8e) induces pronounced surface oxidation within a short duration, as evidenced by the significant formation of ether groups (O–C–O) corresponding to the peak at 286.26 eV (19.37%), indicative of oxidation of pre-existing surface functionalities.^58,60^ The marked increase in carbonyl and carboxyl species further reflects an elevated surface polarity and chemical reactivity, which are conducive to enhanced adhesion performance. Nonetheless, the aggressive nature of such oxidation processes may predispose the material to surface degradation or compromise its structural integrity if exposure times are extended, underscoring the necessity for precise control of plasma treatment conditions.

In the N 1s spectra (Figure 9), the control sample (Figure 9a) shows three peaks: pyridinic-N (sp2 nitrogen: =NH or =N−) at 399.24 eV (54.33%), pyrrolic-N (sp3 nitrogen: C–NH2 or C2–NH) at 399.85 eV (41.79%), and N–O at 401.29 eV (3.88%). After argon plasma treatment (Figure 9b), the pyridinic-N peak drops significantly (∼22%), the pyrrolic-N peak remains nearly unchanged, and the N–O peak increases (∼4%). A new peak at 398.9 eV (18.05%) appears, attributed to amine groups. While argon plasma does not introduce new nitrogen species, it reorganizes or oxidizes existing groups, as shown by increased N–O bonds. Nitrogen plasma at 1200 s (Figure 9c) incorporates new nitrogen functionalities. In addition to pyridinic-N (34.45%) and pyrrolic-N (41.67%), a new peak at 400.43 eV (23.88%) appears, corresponding to tertiary amines or amides. Bai et al.^60^ also associate peaks near 400.6 eV with quaternary nitrogen (N bonded to three carbon atoms in a central position), indicating successful incorporation of nitrogen functionalities onto the composite surface. At 1800 s (Figure 9d), the surface further stabilizes, with a 38.33% increase in the 400.31 eV peak (amides/tertiary amines) and a rise in N–O content (5.10%). For oxygen plasma (Figure 9e), the pyridinic-N peak remains dominant (50.85%), and N–O bonding increases significantly (∼45%) compared to the control, confirming the strong oxidative nature of this plasma, which enhances oxidation of existing nitrogen groups rather than introducing new ones.

High-resolution O 1s spectra analysis (Figure 10) reveals distinct chemical changes on the composite surface following plasma treatments. In the control sample (Figure 10a), three main peaks are observed: a carbonyl-related peak (C=O) at 531.69 eV (46.37%), a peak at 532.32 eV associated with ether (C–O–C) or hydroxyl (O–H) groups (21.56%), and a carboxylic peak (O–C=O) at 532.88 eV (32.07%), characteristic of the epoxy matrix. Following argon plasma treatment at 20 W for 600 s (Figure 10b), a redistribution of oxygen-containing species is observed. The intensity of ether/hydroxyl groups increases by approximately 9%, and a new peak emerges at 533.27 eV, initially attributed to lactone-type structures (O–C­(=O)−). However, as no clear evidence of lactones was detected in the XPS spectrum, this assignment was excluded. Additionally, a ∼4% increase in carbonyl content supports the enhanced oxidation of the surface, contributing to higher surface energy and polarity. For nitrogen plasma treatment at 20 W and 1200 s (Figure 10c), a substantial increase (∼45%) in ether/hydroxyl groups is observed. This is likely due to dation, where the plasma generates reactive surface sites and high-energy species that interact with atmospheric oxygen. Concurrently, carbonyl and carboxylic groups decrease by ∼30 and ∼15%, respectively, suggesting transformation into more stable ether and hydroxyl functionalities.

Extending the nitrogen plasma exposure to 1800 s (Figure 10d) continues the trend of carbonyl depletion (∼39%) and further increases ether/hydroxyl groups (∼40%), albeit less dramatically than at 1200 s. Interestingly, the decrease in carboxylic groups becomes less significant (∼0.5%) compared to the control. This supports the idea that surface oxidation and oxygen functionalization can occur even in oxygen-free plasmas, mediated by post-treatment atmospheric interactions.^61^ Finally, oxygen plasma treatment at 20 W and 600 s (Figure 10e) results in direct oxidation, evidenced by increased carbonyl (∼4%) and ether/hydroxyl (∼9%) groups. In contrast, carboxylic groups decrease by ∼13%, which can be attributed to plasma-induced decarboxylation and etching effects that remove the outermost oxidized layers.^61,62^ Another contributing factor may be the reorganization of the surface toward more thermodynamically stable oxygenated species, such as ethers, which aligns with the observed chemical evolution.^63^

Table 5 summarizes all XPS data obtained from the analyses discussed above, including detailed information on the functional groups containing carbon, oxygen, and nitrogen. This comprehensive overview highlights the chemical modifications on the composite surface induced by the different plasma treatments.

**Table 5:** Atom Composition for Control and Plasma-Treated Samples^a^

			element content (%)				
atom	binding energy range (eV)		control	argon 20 W 600 s	N_2_ 20 W 1200 s	N_2_ 20 W 1800 s	O_2_ 20 W 600 s
C 1s	C–C	284.4–284.7	66.45	68.23	56.88	55.72	63.99
	C–N or C–O	285.2–285.7	30.19	14.61	37.91	38.49	14.22
	O–C–O	286.4–286.8	N.D.	12.94	N.D.	N.D.	19.37
	O–C=O	287.8–288.5	3.44	4.21	5.22	5.78	2.42
N 1s	Pyridine–N	398.5–399.3	54.33	32.46	34.45	56.57	50.85
	Pyrrole–N	399.5–400.2	41.79	41.39	41.67	N.D.	N.D.
	N–H or C–NH–C	400.5–401.5	N.D.	18.05	23.88	38.33	N.D.
	N–O	401.5–402.5	3.88	8.09	N.D.	5.10	49.15
O 1s	C=O	530.8–531.5	46.37	17.81	16.02	7.30	50.38
	O–H or C–O–C	531.5–532.3	21.56	53.63	66.41	61.26	30.20
	O–C=O	532.5–533.0	32.07	16.67	17.56	31.44	19.42
	O–C(=O)– (cyclic)	533.1–533.5	N.D.	11.89	N.D.	N.D.	N.D.

a N.D. = Not Detected.

### 3.4 Surface Morphology Analysis via SEM

The SEM micrographs of the untreated and plasma-treated CFRP samples are presented in Figure 11. The control sample (Figure 11a) exhibits typical characteristics of an untreated CFRP surface: a smooth and homogeneous appearance with small, shallow pores, and carbon fibers covered by the epoxy resin matrix.^64^ After plasma treatment with argon at 20 W for 600 s (Figure 11b), the surface maintains a relatively smooth and homogeneous profile; however, larger and deeper pores begin to appear due to localized etching effects. Argon plasma primarily removes weakly bound surface contaminants and induces slight surface ablation, creating reactive sites with minimal chemical modification. The limited morphological changes observed are thus attributable to mild physical sputtering and selective ablation of the epoxy matrix rather than bulk energy absorption. Following plasma treatment with nitrogen at 20 W for 1200 s (Figure 11c), the surface exhibits more pronounced changes, confirming the trends observed via confocal microscopy. Nitrogen plasma induces etching and introduces reactive species, including oxygen-containing radicals via interaction with ambient air, promoting oxidation and local degradation of the epoxy matrix. This results in deeper and more widely distributed pores. The observed pore distribution reflects both the stochastic nature of plasma-surface interactions and the nonuniform exposure across the sample surface, a characteristic inherent to composite materials. Extending the nitrogen plasma treatment to 1800 s (Figure 11d) further amplifies this effect, consistent with increased interaction time between the CFRP surface and the reactive plasma species.

**Figure 11:**
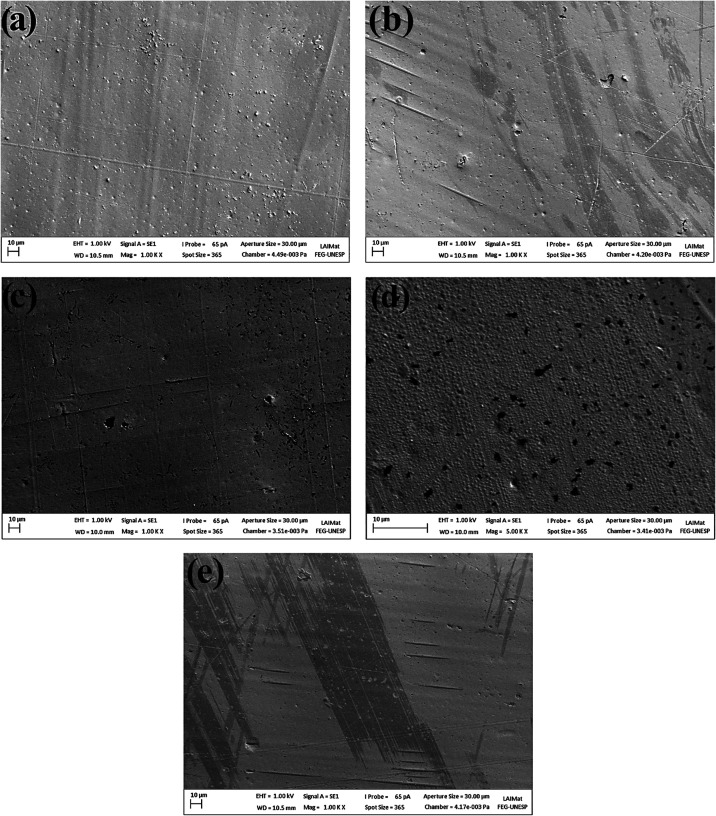
SEM micrographs of: (a) Control sample; and plasma treated samples with (b) Ar–20 W–600 s; (c) N_2_–20 W–1200 s; (d) N_2_–20 W–1800 s; (e) O_2_–20 W–600 s.

In the case of oxygen plasma treatment at 20 W for 600 s (Figure 11e), surface modifications are dominated by direct oxidative etching rather than point defects such as deep pores. The surface exhibits generally increased roughness, with carbon fibers more exposed due to selective resin removal. Localized oxidation events contribute to the formation of a roughened morphology, explaining the differences in surface finish compared to argon or nitrogen plasma treatments. These observations highlight that the combination of oxidative etching and chemical interactions governs the evolution of surface morphology in CFRP, rather than macroscopic energy absorption. The resulting topographical changes, including increased roughness and controlled pore formation, facilitate enhanced interfacial bonding with adhesives and are consistent with reported improvements in mechanical performance.^65–69^

### 3.5 DMA Characterization of Plasma-Activated CFRP

In addition to the physicochemical modifications evaluated in the previous sections, it is essential to assess whether these surface alterations affect the overall viscoelastic behavior of the CFRP. While most studies in the literature focus on improvements in adhesive or structural properties after plasma treatment, the potential impact on the viscoelastic characteristics of the composite remains largely unexplored. In this context, Dynamic Mechanical Analysis (DMA) was conducted to investigate whether different plasma treatments induce significant changes in storage modulus (*E*′), loss tangent (tan δ), and shifts in the glass transition temperature (*T*_g_). The results presented here aim to establish a correlation between plasma-induced surface activation and the global viscoelastic performance of the composite. Figure 12 shows the *E*′ curves, while Figure 13 displays the tan δ curves for all plasma-treated samples.

**Figure 12:**
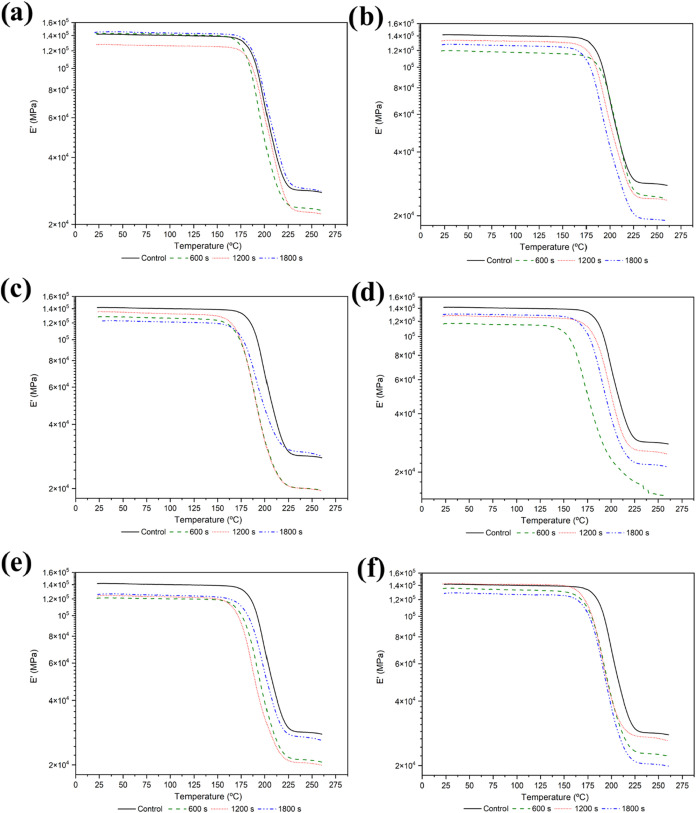
Storage modulus curves for plasma treated samples: (a) Ar–20 W; (b) Ar–50 W; (c) N_2_–20 W; (d) N_2_–50 W; (e) O_2_–20 W; (f) O_2_–50 W.

**Figure 13:**
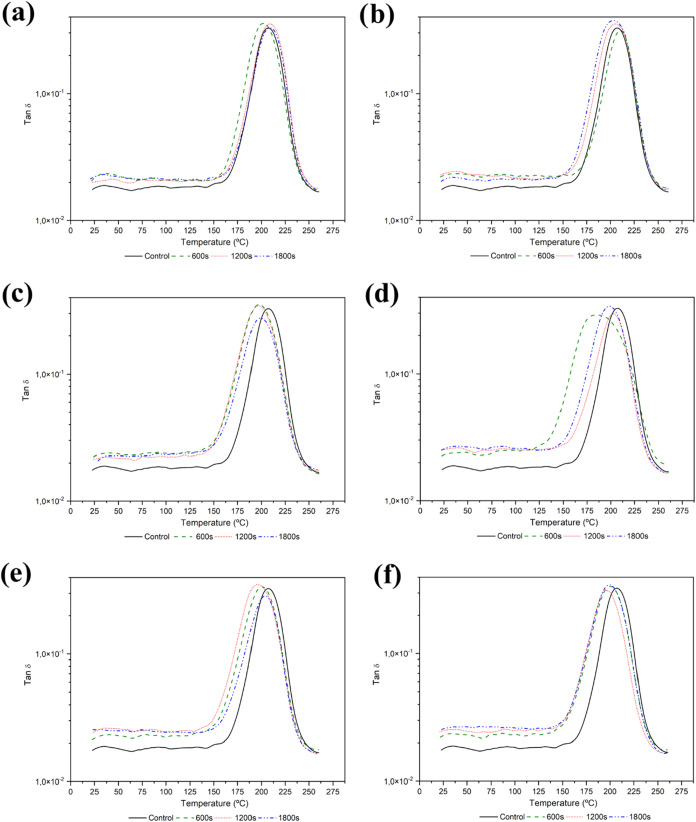
Tan δ curves for plasma treated samples: (a) Ar–20 W; (b) Ar–50 W; (c) N_2_–20 W; (d) N_2_–50 W; (e) O_2_–20 W; (f) O_2_–50 W.

The analysis reveals a general trend of reduced storage modulus at room temperature following plasma treatment. This behavior can be attributed to chemical and structural alterations in the epoxy matrix near the composite surface. Reactive plasmas, particularly those generated with oxygen and nitrogen, can promote localized degradation of the epoxy, including chain scission, network rearrangement, and oxidation.^70,71^ These combined effects result in a slightly weakened surface layer, which may reduce the composite’s stiffness. Indeed, average reductions between 6 and 10% in *E*′ were observed. Similarly, *T*_g_ values derived from the peak of the tan δ curve also showed a decreasing trend, albeit to a lesser extent than *E*′. This decrease may be related to several factors: chain scission and localized oxidation can reduce the average molecular weight and cross-link density of the epoxy, increasing polymer chain mobility; selective removal of less cross-linked or amorphous regions may lead to a more heterogeneous and structurally fragile surface; and the incorporation of polar functional groups may act as localized plasticizers, further enhancing segmental mobility.^72^ On average, *T*_g_ values decreased by 2 to 5% after plasma treatment. A summary of the extracted properties, along with the percentage differences compared to the control sample, is provided in Table 6 for argon plasma, Table 7 for nitrogen plasma, and Table 8 for oxygen plasma. Overall, the reductions observed in *E*′ and *T*_g_ do not constitute critical losses, especially when compared to the mechanical and chemical activation effects provided by plasma treatment. Plasma-induced surface modification significantly enhances interfacial adhesion, resulting in improved mechanical performance of bonded assemblies. These benefits are particularly valuable in structural bonding applications, even between dissimilar materials, where interfacial quality is a key factor. Thus, the practical gains from surface functionalization outweigh the modest viscoelastic variations observed.

**Table 6:** Viscoelastic Properties for Argon Plasma Treated Samples

power (W)	time (s)	*E*′ (MPa) at 30 °C	range of difference versus control (%)	tan δ (°C)	range of difference versus control (%)
20	600	143003.8	0.84	201.1	–2.94
	1200	128315.4	–9.67	208.0	0.39
	1800	145265.2	2.41	209	0.87
50	600	119961.4	–15.43	210.7	1.69
	1200	133653.1	–5.84	204.7	–1.21
	1800	128387.7	–9.49	202.4	–2.32

**Table 7:** Viscoelastic Properties for Nitrogen Plasma Treated Samples

power (W)	time (s)	*E*′ (MPa) at 30 °C	range of difference versus control (%)	tan δ (°C)	range of difference versus control (%)
20	600	128368.4	–9.50	196.7	–5.07
	1200	135447.8	–4.51	196.3	–5.26
	1800	122806.1	–13.42	198.9	–4.01
50	600	116873.9	–17.60	183.3	–11.53
	1200	128541.2	–9.38	203.5	–1.79
	1800	130661.4	–7.88	197.5	–4.68

**Table 8:** Viscoelastic Properties for Oxygen Plasma Treated Samples

power (W)	time (s)	*E*′ (MPa) at 30 °C	range of difference versus control (%)	tan δ (°C)	range of difference versus control (%)
20	600	121341.7	–14.45	199.7	–3.62
	1200	124629.7	–12.14	195.8	–5.50
	1800	126491.0	–10.82	203.6	–1.74
50	600	135816.9	–4.25	199.4	–3.76
	1200	142526.1	+0.48	196.2	–5.31
	1800	129016.2	–9.04	199.1	–3.91

## 4 Conclusion

This study rigorously examined the influence of plasma treatments using argon, nitrogen, and oxygen gases, with exposure times of 600, 1200, and 1800 s, on the surface activation of a CFRP composite intended for structural bonding. Plasma treatment was shown to significantly enhance surface roughness, facilitating mechanical interlocking, and to modify surface chemistry by introducing and rearranging functional groups that promote chemical adhesion. Among argon plasma conditions, treatment at 20 W for 600 s achieved a 16% increase in roughness and improved surface polarity, though hydrophobic recovery was rapid, with a 31% increase in contact angle within 1 h. Nitrogen plasma at 20 W for longer durations (1200 and 1800 s) induced substantial surface topographical changes, including deep pore formation that increased roughness by approximately 140 and 115%, respectively, while introducing high-energy nitrogen functional groups and promoting indirect surface oxidation; hydrophobic recovery was most pronounced, stabilizing at a contact angle of 61° after 12 h. Oxygen plasma at 20 W for 600 s yielded the greatest roughness enhancement (∼200%) and introduced oxygen-containing groups that markedly increased surface polarity; it also exhibited the lowest hydrophobic recovery, with the contact angle stabilizing at 42° after nearly 70 h, a 55% improvement over untreated CFRP. Optimal treatment conditions generally involved low power (20 W) and brief exposure (600 s), except for nitrogen plasma which longer exposure time. XPS analysis confirmed effective surface cleaning through removal of the outermost layer. Dynamic mechanical analysis revealed minor reductions in storage modulus (6–10%) and glass transition temperature (2–5%), indicating increased molecular mobility; however, these slight changes are outweighed by the significant improvements in interfacial adhesion and mechanical performance documented in the literature. These results validate plasma treatment as a clean, rapid, and efficient approach for enhancing CFRP surface properties, supporting its application in structural bonding contexts.
